# From Guidelines to Practice: Embedding MEED Education for Safer Eating Disorder Care

**DOI:** 10.1192/bjo.2026.11299

**Published:** 2026-06-30

**Authors:** Ella McGowan, Emma Barrow, Adelle Williams

**Affiliations:** 1 Birmingham & Solihull Mental Health NHS Foundation Trust, Birmingham, United Kingdom; 2 University Hospitals Birmingham NHS Foundation Trust, Birmingham, United Kingdom

## Abstract

**Aims:**

Medical Emergencies in Eating Disorders (MEED) guidelines provide essential recommendations for identifying and managinghigh-risk eating disorder presentations in acute hospital settings. Local audit data showed inconsistent referral pathways and incomplete documentation of MEED aligned risk assessments, indicating gaps in clinician confidence and understanding. This project aimed to improve the knowledge and confidence of resident doctors, consultant psychiatrists, nurses and allied health professionals (AHPs) in applying MEED guidance, with an emphasis on risk assessment, safe refeeding, legal frameworks, and multidisciplinary collaboration.

**Methods:**

A series of interactive teaching sessions were delivered to diverse clinical groups, including foundation doctors, psychiatry residents, consultant psychiatrists and multidisciplinary liaison psychiatry teams. Teaching was case-based, using real patient scenarios to illustrate MEED risk stratification, indicators for urgent medical admission, safe refeeding principles, decision-making under the Mental Capacity Act (MCA) and Mental Health Act (MHA), and best-practice multidisciplinary management. Participants completed structured pre- and post-session questionnaires evaluating confidence, the relevance of case examples, the likelihood of applying learning, and suggestions for improvement.

**Results:**

A total of 50 clinicians completed the pre-session questionnaire, and 67 completed the post-session questionnaire. At baseline, 68% reported low confidence. There was wide variation in clinical experience, with 32% having never encountered patients with eating disorders.

Following the teaching, most participants reported improvements in confidence, with 76% reporting “significant” or “quite a bit” of improvement, frequently citing clearer understanding of MEED principles, including “*MEED guidelines and applying it to patients*”, as well as practical skills such as “*give thiamine before glucose*”. 97% of participants indicated that they are “very likely” or “somewhat likely” to implement the learning in their practice. Post-session, clinicians expressed a stronger grasp of legal frameworks, noting improved confidence in “*treatment under MCA*” and recognising the need to “*always assess for capacity in patients refusing treatment*”. The importance of coordinated multidisciplinary practice was repeatedly highlighted by clinicians, noting that “*managing patients jointly with physicians will produce the best result*” and that effective care relies on close “*interaction between medical team, PLT and dieticians*”.

**Conclusion:**

Interactive, case-based MEED teaching is an effective educational approach that improves clinicians’ confidence and readiness to manage eating-disorder emergencies. Embedding MEED teaching within routine induction and MDT training, alongside accessible reference materials, may strengthen clinical practice and patient safety across acute care settings. The next steps involve training physicians in accident and emergency departments and in acute medical wards.

